# Downregulation of ITGβ3 in colon adenocarcinoma reveals poor prognosis by affecting genome stability, cell cycle, and the tumor immune microenvironment

**DOI:** 10.3389/fonc.2022.1047648

**Published:** 2023-01-20

**Authors:** Lei Zhao, Xiaoting Ma, Guangxin Li, Pengfei Zhao, Haishan Lin, Yingjie Ma, Huihui Li, Jing Yu

**Affiliations:** ^1^ Cancer Center, Beijing Friendship Hospital, Capital Medical University, Beijing, China; ^2^ Radiation Oncology, Beijing Tsinghua Changgung Hospital, School of Clinical Medicine, Tsinghua University, Beijing, China

**Keywords:** ITGβ3, tumor invasion, prognosis, COAD, bioinformatics analysis

## Abstract

**Introduction:**

Abnormal expression of integrin subunit beta 3 (ITGβ3), a gene-encoding protein, is related to the occurrence and development of cancers; however, the biological role of ITGβ3 in colon adenocarcinoma (COAD) remains unclear.

**Methods:**

We used the Cancer Genome Atlas database to obtain the clinical data of patients with COAD, analyzed the mRNA gene clusters related to ITGβ3, and analyzed the interaction signal pathway and interaction protein network of the differentially expressed gene clusters. The results showed that ITGβ3 expression in COAD tumor tissues was significantly downregulated compared with that in paracancerous tissues. Low ITGβ3 expression in tumor tissues is associated with poor overall survival of patients with COAD. In multivariate analysis, stage IV and ITGβ3 low expression were independent prognostic factors. Gene Ontology analysis showed that differentially expressed genes (DEGs) were significantly enriched in leukocyte migration, cell adhesion, and extracellular matrix (ECM) organization. Kyoto Encyclopedia of Genes and Genomes analysis revealed that the DEGs were mainly enriched in ECM-receptor interactions, focal adhesion, and the PI3K-Akt signaling pathway. Protein-protein interaction network analysis revealed the hub and seed genes of the key modules related to ITGβ3. Finally, we analyzed the correlation between TGβ3 and immune-related genes and found that ITGβ3 expression was significantly correlated with tumor purity and infiltration level of dominant immune cells.

**Discussion:**

These findings indicate that ITGβ3 downregulation in COAD may profoundly affect genome stability and multiple steps of the cell cycle, alter the tumor immune microenvironment, and be related to the prognosis of patients with COAD.

## 1 Introduction

Colorectal cancer (CRC) is the third most common malignant tumor and second leading cause of cancer-related deaths worldwide. Its morbidity and mortality rates have increased rapidly in recent years (1). Colon adenocarcinoma (COAD) is an important type of CRC. The incidence rate of COAD is 2.5 times that of rectal cancer (2). Progress has been made in improving the early diagnosis rate of COAD and reducing its mortality rate, owing to improvements in imaging diagnosis technology and the development of new therapeutic drugs and methods. Nevertheless, the prognosis of many patients with advanced stages of the disease is still very poor, especially for those who already have distant metastases. In these advanced patients, growing evidence suggests that COAD enhances metastatic ability and accelerates disease progression through changes in the tumor microenvironment (TME). The TME, including fibroblasts, immune cells, blood and lymph vessels, and the extracellular matrix (ECM), plays an important role in regulating cancer growth and the therapeutic response through cytokines, cell-cell interactions, and mechanistic induction (3, 4). Cancer-associated fibroblasts, one of the most abundant cell types in the tumor stroma, can promote tumor growth and invasion and confer chemotherapeutic resistance (5–7). Cancer-associated fibroblasts can trigger the migration and invasion of cancer cells by integrating ECM protein-fibronectin or by regulating intracellular signaling pathways through integrins (5, 6, 8).

Integrins are cell adhesion molecules containing a/b heterodimers that mediate cell-cell, cell-ECM, and cell-pathogen interactions. Integrins are involved in signal transduction, cell migration, and proliferation (9). Integrin subunit beta 3 (ITGβ3) is a receptor for various proteins such as fibronectin, laminin, matrix metalloproteinase-2, osteomodulin, and vitronectin. ITGβ3 is highly expressed in various malignant tumors; however, in COAD cells, the role of ITGβ3 is debated. Although ITGβ3 has been reported to be necessary for the survival, invasion, and migration of colon cancer cells (10–12), ITGβ3 can also inhibit HER-2 signal transduction and exert a tumor suppressor-like activity in colon cancer cells. Regarding the prognostic significance of ITGβ3 in COAD, ITGβ3 expression was significantly increased in the cytoplasm of cancer cells (13). In contrast, another study showed that ITGβ3 gene and protein levels were only elevated in mucinous carcinoma but not in common COAD (14).

Another article published regarding ITGβ3 contrasted conventional views, which suggested that conditioned medium treated with ITGβ3-deficient pericytes could significantly increase the number of tumor cells, suggesting that ITGβ3 may regulate tumor cell growth *via* paracrine pathways (15).

Thus, the objective of the current study was to investigate the expression and mutation of ITGβ3 in patients with COAD, assess the genomic changes and functional networks associated with ITGβ3 in COAD, and discuss its role in tumor immunity.

## 2 Materials and methods

### 2.1 Study cohort

Gene expression quantification, miRNA expression quantification data, and clinical data of tumor tissues and paracancerous tissues were obtained from the COAD dataset of the Cancer Genome Atlas (TCGA) database (https://portal.gdc.cancer.gov/), and the gene expression matrix was acquired after the formalin-fixed paraffin-embedded tissue sample (which has been proven to be ineffective for sequencing analysis) and low expression genes were removed (average expression level < 1). Using the R-package edgeR, we performed differentially expressed genes (DEGs) of mRNA and miRNA expression between tumor and non-tumor samples with threshold parameters defined as |log_2_FC| > 1 and FDR < 0.05 (FC: fold change; FDR: false discovery rate) (16). The meta-analysis of ITGβ3 expression in tumor and paracancerous tissues in different studies on COAD was performed using Oncomine (**Supplementary Table 1**). All datasets used in this study were downloaded from the public TCGA database, which allows researchers to download and analyze public datasets for scientific purposes; thus, ethical approval was not required.

### 2.2 Survival analysis

The clinical and ITGβ3 RNA data of patients with COAD were downloaded from TCGA. The cutoff value for ITGβ3 expression was determined by its median value. Patients were classified into the low expression group (below the median) and high expression group (above the median) to analyze the correlation between ITGβ3 expression and survival rates. Clinicopathological characteristics associated with overall survival in patients data in TCGA using Cox regression and the Kaplan-Meier method. Kendall’s tau-b (K) correlation method was used to analyze the correlation between the expression of ITGβ3 and clinical characteristics (sex and stage). Survival analyses were performed using R-packages survival(version 3.2.7) and survminer(version 0.4.8). HR > 1 and HR < 1 indicated negative and positive correlations, respectively. Statistical significance was set at p < 0.05.

### 2.3 Immune correlation analysis

ITGβ3 expression in COAD and its correlations with the abundance of immune infiltrates, including B cells, CD4+ T cells, CD8+ T cells, neutrophils, macrophages, and dendritic cells, and tumor purity were analyzed using the Tumor Immune Estimation Resource (TIMER) database. Correlations between the DEGs and immune-related genes from the ImmPort database were analyzed (**Supplementary Table 1**).

### 2.4 Bioinformatics analysis

Gene Ontology (GO) enrichment analysis and Kyoto Encyclopedia of Genes and Genomes (KEGG) pathway analysis of genes related to ITGβ3 were conducted using the Database for Annotation, Visualization, and Integrated Discovery (DAVID; **Supplementary Table 1**). The genes related to ITGβ3 were uploaded to the STRING website to analyze the interactions between these proteins (**Supplementary Table 1**). The minimum required interaction score was set to 0.400 (medium confidence), and protein nodes that did not interact with other proteins were removed. The Molecular Complex Detection (MCODE) plug-in in Cytoscape was used to identify functional molecular complexes in the protein–protein interaction (PPI) network, and a MCODE score higher than 5.0 was considered significant. The Cytohubba plug-in was used to select the top ten hub genes in the PPI network. The correlation between DEGs and ITGβ3 was analyzed using the Spearman correlation analysis method. According to conventional practice, r ≥ 0.8 indicates a high correlation between the expression of two genes; when 0.5 ≤ |r| < 0.8, it indicates a moderate correlation; and when 0.3 ≤ |r| < 0.5, the correlation is low. To obtain all relevant genes for PPI network analysis, r ≥ 0.5 was regarded as relevant in this study.

### 2.5 Statistical analysis

The difference between the two groups was tested using a two-tailed Student’s paired or unpaired t-test. A p-value < 0.05 was considered as the threshold. The log-rank test p < 0.05 indicated significant differences in survival time.

## 3 Results

A total of 514 samples were downloaded, including 473 tumor and 41 paracancerous tissues. Survival information in TCGA-COAD of 336 patients was obtained from a clinical dataset. A total of 456 miRNA expression quantification samples (448 tumor tissues and 8 paracancerous tissues) were used for differential expression analysis.

### 3.1 ITGβ3 expression comparison

**Figure 1:**
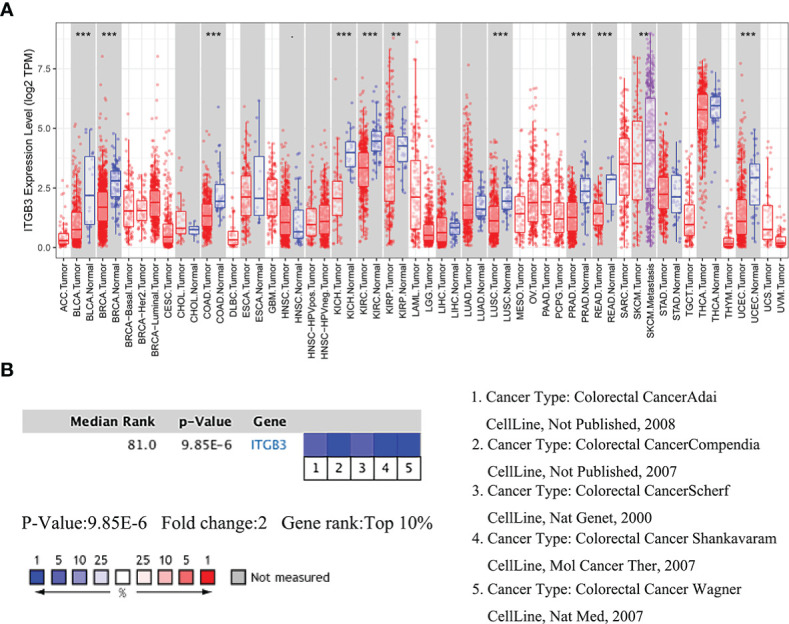
Comparison of ITGβ3 mRNA expressions in Oncomine. **(A)** Expressions of ITGβ3 in different tumors. **(B)** Meta-analysis of ITGβ3 expression in five analyses. *, p < 0.05, **, p < 0.01, ***, p < 0.001.

Analysis of the COAD cohorts in TCGA revealed that ITGβ3 mRNA expression was significantly lower in COAD tissues than in paracancerous tissues (**Figure 1A**). The median ITGβ3 expression in paracancerous tissues was 174.00 (129.00–324.00), while in tumor tissues it was 54.00 (31–114.00). ITGβ3 was a low-expression gene (p = 9.85E-6) and ranked in the top 10% of Oncomine (**Figure 1B**, **Supplementary Table 1**).

Univariate analysis of the correlation using Cox regression revealed that low expression of stage IV and ITGβ3 was significantly associated with overall survival. Kendall’s tau-b (K) correlation coefficient analysis showed that only the tumor stage was weakly correlated with ITGB expression (r = 0.101, p = 0.046). In multivariate analysis, stage IV and ITGβ3 low expression were independent prognostic factors (**Table 1**).

**Table 1:** (A) Association with overall survival and clinicopathologic characteristic in patient data from TCGA using Cox regression.

Clinical characteristics	Median(Q1-Q3)	HR(95%CI)	p- Value
A.			
Age			
21-40 Yrs	106.00(54.00-137.50)	1	
41-60 Yrs	70.00(32.00-132.00)	0.26(0.07-1.94)	0.052
61-80 Yrs	53.50(31.75-132.25)	0.66(0.20-2.17)	0.491
81-100 Yrs	59.00(33.50-105.00)	0.73(0.18-2.93)	0.656
Gender			
Female	55.00(32.00-122.75)	1	
Male	64.00(31.75-132.25)	1.42(0.81-2.50)	0.227
Stage			
Stage I	51.00(32.00-114.00)	1	
Stage II	53.50(32.25-106.75)	1.15(0.33-34.04)	0.829
Stage III	69.00(29.00-141.00)	1.32(0.37-4.66)	0.670
Stage IV	69.50(41.00-159.50)	5.26(1.55-17.91)	0.008
ITGβ3(High expression)	128.50(93.50-203.25)	1	
ITGβ3(Low expression)	32.00(21.75-42.25)	2.00(1.09-3.64)	0.020
B.			
Stage IV	69.50(41.00-159.50)	7.07(2.06-24.22)	0.001
ITGβ3(Low expression)	32.00(21.75-42.25)	2.59(1.38-4.86)	0.003

(B) Multivariate survival using Cox regression.

### 3.2 ITGβ3 expression is survival-associated

**Figure 2:**
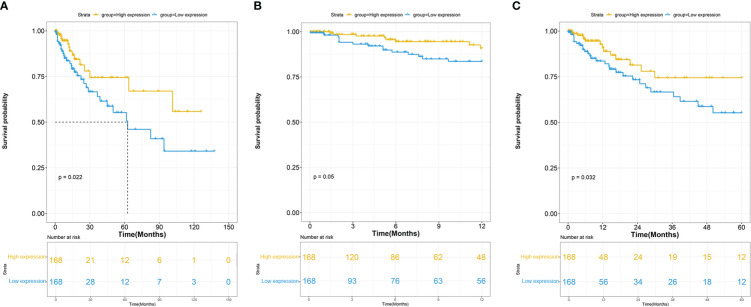
**(A)** Overall survival (OS) of COAD patients grouped by ITGβ3 median cutoff in TCGA; one-year **(B)** and five-year **(C)** OS in comparison between high- and low-ITGβ3 groups.

The association between ITGβ3 expression and survival outcome in the COAD cohort was evaluated using the Kaplan-Meier survival curve (**Figure 2**). The patients were separated into two groups according to the median ITGβ3 expression level. In the high and low expression groups, there were 93 males (55.35%) and 87 males (51.78%), respectively. In the high expression group, ages 21–40 accounted for 4.76% (8/168), 41–60 accounted for 30.95% (52/168), 61–80 accounted for 51.79% (87/168), and 81–100 accounted for 12.50% (21/168). In the low expression group, ages 21–40 accounted for 2.38% (4/168), 41–60 accounted for 29.17% (49/168), 61–80 accounted for 55.36% (93/168), and 81–100 accounted for 13.09% (22/168) (**Table 2**).

**Table 2:** Associations with overall survival and expression of ITGβ3 in patient data from TCGA using Cox regression.

Clinical characteristics		High expression (%)	Low expression (%)
Gender	Female	75 (44.65)	81 (48.22)
	Male	93 (55.35)	87 (51.78)
	Total	168 (100)	168 (100)
Age	21-40 Yrs	8 (4.76)	4 (2.38)
	41-60 Yrs	52 (30.95)	49 (29.17)
	61-80 Yrs	87 (51.79)	93 (55.36)
	81-100 Yrs	21 (12.50)	22 (13.09)
	Total	168 (100)	168 (100)
HR (95%CI), p	1-year OS	2.29 (1.00-5.37), 0.050	
	5-year OS	1.99 (1.06-3.79), 0.032	
	OS	2.00 (1.10-3.64), 0.022	

These results indicate that low ITGβ3 expression in tumor tissues was considerably associated with poor overall survival of patients with COAD (log-rank p = 0.022, HR = 2.00 (1.10–3.64); **Figure 2A**). Subgroup analysis revealed that the downregulation of ITGβ3 in tumor tissues was a risk factor for reduced one-year Overall Survival(OS) (log-rank p = 0.050, HR = 2.29 (1.00–5.37); **Figure 2B**) and five-year OS (log-rank p = 0.032, HR = 1.99 (1.06–3.79); **Figure 2C**) in patients with COAD (**Table 2**).

### 3.3 ITGβ3 co-expression networks in COAD

Patients with COAD were separated into two groups according to the median ITGβ3 expression level. DEGs were identified using the R package(version 3.6.3) ‘edgeR’ (17) from the Bioconductor project. A total of 702 common DEGs were detected in COAD tissues, including 62 downregulated and 640 upregulated genes.

### 3.4 KEGG and GO pathway enrichment analyses

GO analysis showed that ITGβ3-related genes were significantly enriched in ECM organization, cell adhesion, and leukocyte migration (BP level), plasma membrane, cell surface, and extracellular exosome (CC level), protein binding, protease binding, and receptor activity (MF level). KEGG pathway analysis showed that ITGβ3 interactive genes were significantly enriched in ECM-receptor interaction, focal adhesion, and the PI3K-Akt signaling pathway (**Table 3**).

**Table 3:** GO and KEGG pathway enrichment analysis of DEGs in COAD samples.

Term	Description	Count in gene set	FDR	P-value
GO:0030198	Extracellular matrix organization	58	9.41E-35	3.46E-38
GO:0007155	Cell adhesion	76	2.04E-28	1.50E-31
GO:0050900	Leukocyte migration	20	5.32E-06	1.76E-08
GO:0005886	Plasma membrane	213	4.39E-12	6.00E-14
GO:0009986	Cell surface	54	4.28E-11	7.01E-13
GO:0070062	Extracellular exosome	156	1.94E-10	3.71E-12
GO:0005515	Protein binding	339	1.11E-04	1.90E-06
GO:0002020	Protease binding	13	0.004693	1.03E-04
GO:0004872	Receptor activity	19	0.010429	2.61E-04
hsa04512	ECM-receptor interaction	26	6.62E-14	3.54E-16
hsa04510	Focal adhesion	37	3.13E-13	3.34E-15
hsa04151	PI3K-Akt signaling pathway	45	1.89E-11	3.03E-13

### 3.5 ITGβ3 co-expression derived PPI network

**Figure 3:**
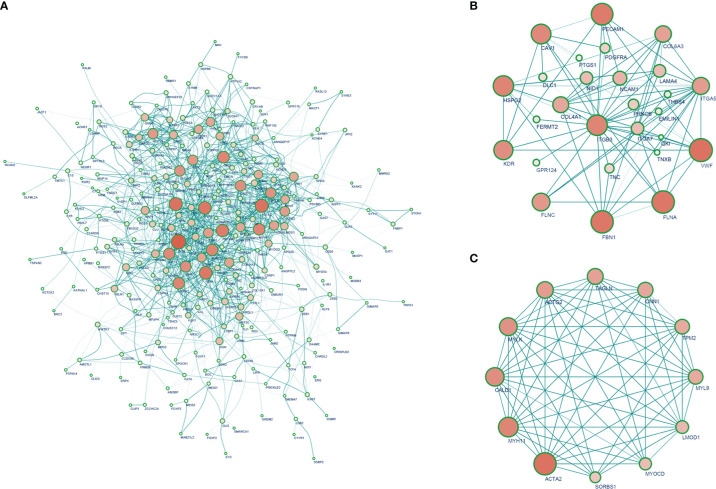
**(A)** PPI network of strong ITGβ3 co-expression genes. **(B)** PPI network that interacts with ITGβ3. **(C)** Top 10 hub genes.

A total of 326 highly correlated genes (Spearman’s correlation coefficient > 0.5) were selected from the DEGs. To identify the significant modules, we merged the 326 DEGs in the STRING online database and Cytoscape 11.0 (**Supplementary Table 1**). PPI networks of DEGs (**Figure 3A**) and ITGβ3 (**Figure 3B**) were constructed. The three most significant modules (MCODE > 5) were obtained using the MCODE plug-in app in Cytoscape. The seed genes tropomyosin 2 (TPM2), G protein subunit alpha O1 (GNAO1), and chordin-like 1 (CHRDL1) were all significantly and positively correlated with ITGβ3 (**Supplementary Figure 1**, **Supplementary Table 2**).

The 10 hub genes significantly positively correlated with ITGβ3 were filtered according to node degrees on the CytoHubba plug-in Cytoscape, including ACTA2, MYH11, MYLK, MYL9, ACTG2, CALD1, LMOD1, TAGLN, CNN1, and TPM2 (**Figure 3C**, **Supplementary Table 2**).

### 3.6 ITGβ3 co-expression miRNAs in COAD

A total of 147 common miRNA DEGs in COAD tissues were obtained, including 91 downregulated genes and 56 upregulated genes. The top five upregulated genes were mir-374a, mir-101, mir-141, mir-182, and mir-142, and the top five downregulated genes were let-7b, let-7d, mir-125a, mir-99b, and mir-150. It is traditionally believed that mir-143 is a tumor suppressor gene, but in this study, we found that mir-143 was upregulated; therefore, the function and role of mir-143 in tumorigenesis and development in COAD still needs to be further studied.

We analyzed the correlation between miRNA and ITGβ3 gene expression using the Spearman analysis method. The results showed that the correlation coefficient between eight miRNAs and ITGβ3 expression was greater than 0.5 (mir-143, mir-133a, mir-132, mir-140, mir-139, mir-490, let-7c, and let-7e) (**Supplementary Table 3**).

### 3.7 Correlation analysis between ITGβ3 and immune related genes

**Figure 4:**
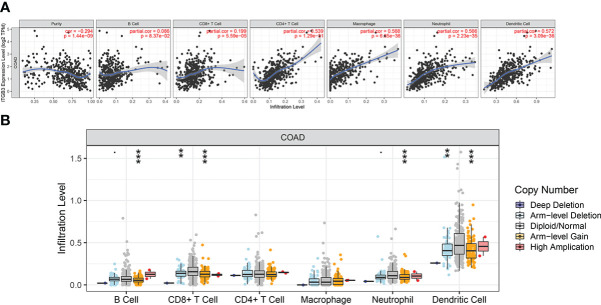
Correlations of ITGβ3 expression with immune infiltration level in COAD. **(A)** ITGβ3 expression significantly related to tumor purity and to infiltrating levels of CD8+ T cells, CD4+ T cells, macrophages, neutrophils, and dendritic cells in COAD. **(B)** ITGβ3 CNV affecting the infiltrating levels of CD8+ T cells, B cells, neutrophils, and dendritic cells in COAD. *, p < 0.05, **, p < 0.01, ***, p < 0.001.

The Estimation of STromal and Immune cells in MAlignant Tumor tissues using Expression data (ESTIMATE) algorithm uses the gene enrichment analysis of a single sample and the gene expression profile of immune cells and stromal cells to infer the degree of infiltration of stromal and immune cells in tumor samples and estimate the purity of tumor cells based on the empirical cumulative distribution function (**Supplementary Table 1**).We investigated whether ITGβ3 expression was correlated with immune infiltration level in patients with COAD *via* the ESTIMATE algorithm. The results showed that ITGβ3 expression was significantly correlated with the ESTIMATE Score (r = 0.514, p = 3.93E-25), Immune Score (r = 0.349, p = 1.48E-11), and tumor purity (r = -0.547, p = 7.58E-29). We also analyzed the relationship between ITGβ3 expression and immune infiltration level using the TIMER online database, and the results showed that ITGβ3 expression was significantly correlated with tumor purity (r = -0.294, p = 1.44E-09) and the infiltration level of dominant immune cells (**Figure 4A**). In particular, ITGβ3 was significantly correlated with the infiltrating levels of B cells, CD8 + T cells, B cells, neutrophils, and dendritic cells (**Figure 4B**).

A total of 2484 immune-related genes were obtained from the Immport database (**Supplementary Table 1**). Of these, 62 immune-related genes were identified as DEGs (**Supplementary Table 4**). There were 15 genes with a correlation coefficient of ITGβ3 greater than 0.7, including OSMR, S1PR1, AKT3, FGF7, ANGPTL2, NRP1, NRP2, IL6ST, IL1R1, PDGFRB, INHBA, CMKLR1, CALCRL, and LTBP2 (**Table 4**).

**Table 4:** Correlation analysis between ITGβ3 and immune-related genes (Cor > 0.7).

Category	Gene Symbol	Gene Name	None		Purity	
			Cor	P	Cor	P
BCRSignalingPathway	AKT3	AKT serine/threonine kinase 3	0.7744	1.10E-92	0.7517	5.01E-75
Chemokine_Receptors	CMKLR1	Chemerin chemokine-like receptor 1	0.7208	1.32E-74	0.6451	3.71E-49
	ANGPTL2	Angiopoietin like 2	0.7561	5.52E-86	0.7299	9.85E-69
	CALCRL	Calcitonin receptor like receptor	0.7061	2.30E-70	0.6798	2.34E-56
	IL1R1	Interleukin 1 receptor type 1	0.7477	4.20E-83	0.7308	5.58E-69
	KDR	Kinase insert domain receptor	0.7279	9.57E-77	0.7024	1.27E-61
	NRP1	Neuropilin 1	0.7522	1.18E-84	0.7207	2.94E-66
	NRP2	Neuropilin 2	0.7258	4.31E-76	0.6891	1.80E-58
	OSMR	Oncostatin M receptor	0.8231	4.04E-114	0.8072	1.61E-94
	S1PR1	Sphingosine-1-phosphate receptor 1	0.7970	6.69E-102	0.7750	1.54E-82
Cytokines	FGF7	Fibroblast growth factor 7	0.7594	3.78E-87	0.6158	9.42E-44
	LTBP2	Latent transforming growth factor beta binding protein 2	0.7073	1.08E-70	0.6693	4.44E-54
	PDGFRB	Platelet derived growth factor receptor beta	0.7370	1.33E-79	0.7005	3.70E-61
Interleukins	IL6ST	Interleukin 6 signal transducer	0.7521	1.33E-84	0.7426	2.55E-72
TGFb_Family_Member	INHBA	Inhibin subunit beta A	0.7267	2.30E-76	0.6900	1.14E-58

### 3.8 Correlation analysis between ITGβ3 and ITGα genes

We also performed a correlation analysis of the ITGα genes that were most closely related to ITGβ3. The top three ITGα genes with the highest correlation with ITGβ3 were ITGα5 (r = 0.773), ITGαX (r = 0.663), and ITGα1 (r = 0.659) (all p < 0.01; **Supplementary Figure 2**).

## 4 Discussion

ITGβ3, also known as CD61 or GP3A, is one of the most widely studied members of the integrin family and plays diverse crucial roles in tumor malignant progression and tumor microenvironment reprogramming (18). Studies have confirmed that ITGβ3 plays an important role in cancers such as breast, gastric, nasopharyngeal, and CRC (10, 19–21). However, the role of ITGβ3 in tumorigenesis and development is debated, and most studies believe that ITGβ3 plays an important role in promoting tumor occurrence and development (18). antitumor drugs targeting ITGβ3 have been developed; however, some studies have shown that ITGβ3 loss leads to rapid tumor growth (15). COAD is a relatively refractory type of tumor. Recently, research on anti-COAD treatments has progressed slowly. As a new type of drug, immune checkpoint inhibitors have not achieved ideal efficacy for the treatment of COAD. Therefore, it is very important to find new targets in the treatment of COAD, especially the integrin family, as existing studies have not explored the correlation of ITGβ3 gene expression in COAD with clinical data, and there is insufficient data to illustrate the prospects of anti-ITGβ3 drugs in the treatment of COAD. To better understand ITGβ3 in COAD and its potential functions in the network, we conducted a bioinformatics analysis of public data to guide future research.

In contrast to the traditional idea that ITGβ3 is highly expressed in tumor tissues, transcriptome analysis of 336 clinical samples from TCGA showed that ITGβ3 mRNA and CNV levels in COAD were significantly lower than those in paracancerous tissues. Analysis of expression changes demonstrated that ITGβ3 expression in tumor tissue decreased sharply upon tumor occurrence but gradually increased with tumor development. Low ITGβ3 expression was significantly associated with low survival rate. Patients at an advanced stage versus those at an early stage and patients with low versus high ITGβ3 expression may have a higher risk. Univariate and multivariate COX regression analyses revealed that ITGβ3 was an independent prognostic factor for COAD. Therefore, downregulation of ITGβ3 as a potential diagnostic and prognostic marker warrants further clinical verification.

Our conclusions were confirmed by a previous study, in which immunohistochemical staining showed that ITGβ3 expression in COAD tissues was lower than that in paracancerous tissues of 49 patients with COAD. In patients with lymph node metastasis, ITGβ3 expression in COAD tissues and epithelial cells of paracancerous tissues was significantly lower than that in the lymph nodes. These results indicate that the expression and role of ITGβ3 in COAD warrants further study (22).

ITGβ3, a marker of angiogenesis, participates not only in the key steps of tumor angiogenesis by regulating cell-cell and cell-matrix interactions, but also in multiple signaling pathways (23). For example, in matrix remodeling, the ECM contains virtually all cytokines, growth factors, and hormones secreted by stromal and tumor cells, which are largely heterogeneous and complex. Previous studies have found that ECM1a strongly induces the expression of the splicing factor heterogeneous nuclear ribonucleoprotein L-like (hnRNPLL) protein of mRNA precursors. By knocking down the expression of hnRNPLL at different levels, we found that the mRNA levels of each subtype of ECM1 were highly stable, indicating that hnRNPLL selectively spliced mRNA to generate ECM1a instead of regulating the expression of the ECM1 subtype by regulating the stability of mRNA. Therefore, the role of integrins in tumor development may not be generalizable. Integrins may affect tumors differently depending on the tissue and organ, and the role of the integrin family in RNA splicing is worthy of further study.

Wu and Zhong et al. (19) have a differing view of ITGβ3. They suggest that a conditioned medium treated with ITGβ3-deficient pericytes can significantly increase the number of tumor cells, suggesting that ITGβ3 may regulate tumor cell growth through paracrine pathways. They further showed that the expression level of the tyrosine kinase FAK was significantly upregulated in pericytes lacking ITGβ3; the highly expressed FAK phosphorylates its substrates HGFR and Akt (S473 and S536) to activate the Akt signaling pathway. p-HGFE and p-Akt further activate the downstream NF-kB signaling pathway, upregulate the phosphorylation level of p65, and ultimately upregulate the expression of the cytokines sICAM-1, IL-1B, CXCL1, CCL2, and TIMP-1. The cytokines secreted from pericytes bind to the ICCR2 receptor on the surface of tumor cells, increasing the phosphorylation level of MEK1/2 in tumor cells, activating the MAPK signaling pathway, and promoting the growth of tumor cells. Therefore, further research is needed to elucidate the function and role of ITGβ3.

The TME involves non-cancer cells in and around the tumor and greatly influences the genome analysis of tumor samples. Because gene dynamics affect genetic diversity and micro-environmental processes, a co-occurrence analysis was performed. TIMER analysis showed that ITGβ3 expression was significantly correlated with tumor purity and the level of dominant immune cell infiltration. In particular, ITGβ3 CNV was significantly correlated with the infiltration levels of B cells, CD8 + T cells, neutrophils, and dendritic cells. Tumor-infiltrating B cells most likely contribute to tumor control through a dual mechanism of (I) antigen presentation and (II) antitumor IgA production in several tumor entities, including COAD (24). The low expression of ITGβ3 in COAD tissues and its correlation with tumor purity and immune cell infiltration also initially revealed the possible reasons for the poor immunotherapeutic effect on COAD. However, further research is needed to clarify whether ITGβ3 is a key factor in mediating T cell therapy.

In addition, 14 immune genes correlated with ITGβ3, with a coefficient of more than 0.7, and the most correlated gene was Homo sapiens oncostatin M receptor (OSMR). OSM from the IL-6 family is mainly produced by activated monocytes and lymphocytes. This multifunctional cytokine can be found in various tumor cells such as in breast cancer, ovarian cancer, lung cancer, melanoma, and osteosarcoma. Our results are consistent with those of other studies showing that macrophage-secreted OSM stimulates inflammatory gene expression in cancer-associated fibroblasts, which in turn induces a pro-tumorigenic environment and engages tumor cell survival and migratory signaling pathways (25). OSMR is under-expressed in COAD tissues due to hypermethylation, whereas it is highly expressed in paracancerous colorectal mucosa (hypomethylation) (26). Similarly, OSMR may be a tumor suppressor gene for COAD, and gene silencing caused by OSMR methylation may be critical for the development of COAD (27). Increased OSMR signaling may favor the establishment of mesenchymal tumors in patients with Inflammatory bowel disease (28). Compared with ITGβ3, these genes may play a vital role in tumor occurrence and development, but there is a significant correlation between ITGβ3 expression and survival rate, which was not observed in the other genes above. The function and role of ITGβ3 in tumorigenesis and development require further study.

With the development of pharmacological research on ITGβ3, inhibitors targeting ITGβ3 have been studied, and some have been put into clinical trials, including cilengitide (29) (CLG, EMD 121974), which is a cyclic RGD peptide that specifically blocks the αv subunit of integrins and is highly specific to αvβ3 integrins (30). Existing research shows that CLG inhibits the binding of αvβ3 integrin to the ECM and prospectively demonstrates antiangiogenic and antitumor effects in many cancers (31). CLG can also promote the detachment of glioblastoma and mesothelioma cells from ECM components through exposed RGD sequences, resulting in anoikis-dependent apoptosis and inhibition of invasion (32). Notably, as an αvβ3 integrin-specific inhibitor, CLG also shows encouraging outcomes in pancreatic cells, gliomas, and some metastatic solid tumors, according to some clinical trials (33–35). However, a further large clinical study indicated that adding CLG to temozolomide chemoradiotherapy does not improve outcomes (36). The limitations of the present study were that none of these findings had biological validation *in vitro*.

## 5 Conclusions

Downregulation of ITGβ3 may serve as a biomarker for the development of COAD, showing profound effects on genome stability, multiple steps in the cell cycle, and immune regulation. Since our findings are inconsistent with the traditional view that ITGβ3 is upregulated in cancers, and since the current clinical efficacy of ITGβ3-targeted drugs is uncertain, it is not recommended to try ITGβ3-targeted drugs in COAD. Nevertheless, our findings need to be further confirmed by large-scale genomic studies of COAD.

## Data availability statement

The original contributions presented in the study are included in the article/**Supplementary Material**. Further inquiries can be directed to the corresponding authors.

## Author contributions

All authors listed have made a substantial, direct, and intellectual contribution to the work and approved it for publication.
