# Supplementary material for: Downregulation of ITGβ3 in colon adenocarcinoma reveals poor prognosis by affecting genome stability, cell cycle, and the tumor immune microenvironment
Source: Front Oncol. 2023 Jan 20;12:1047648. doi: 10.3389/fonc.2022.1047648 (PMC9895777; doi:10.3389/fonc.2022.1047648)
Supplement: Supplementary file 1 [file Table_1.docx]

Supplementary Material

Downregulation of ITGβ3 in colon adenocarcinoma reveals poor prognosis by affecting genome stability, cell cycle, and the tumor immune microenvironment

Lei Zhao^1^, Xiaoting Ma^1^, Guangxin Li^2^, Pengfei Zhao^1^, Haishan Lin^1^,Yingjie Ma^1^,Huihui Li^1*^ and Jing Yu^1*^

*** Correspondence:** Jing Yu Email:yujing026@163.com

Huihui Li Email:huihuimuzi2008@126.com

Supplementary Table 1. Online database for analysis

| **Database** | **URL** | **Introduction** |
| --- | --- | --- |
| Oncomine | <https://www.oncomine.org/> | The expression level of the ITGB3 gene in colorectal cancers was examined in the Oncomine 4.5 database. Oncomine is a cancer microarray database and web-based data-mining platform. The threshold was determined based on the following values: p value of 0.05, and the fold change of 2. |
| DAVID | [http://david.ncifcrf.gov](http://david.ncifcrf.gov/) | Annotation, visualization and integrated discovery database (version 6.8) DAVID is an online function annotation tool. DAVID was used to gene ontology (GO) enrichment analysis, including biological processes (BP), cell components (CC) and molecular functions (MF). The DAVID database is also used to perform pathway enrichment analysis. The threshold was P<0.05. |
| UALCAN | [http://ualcan.path.uab.edu](http://ualcan.path.uab.edu/) | UALCAN uses TCGA level 3 RNA-seq and clinical data from 31 cancer types, allowing analysis of relative expression of genes across tumor and normal samples, as well as in various tumor sub-groups based on individual cancer stages, tumor grade or other clinicopathological features. |
| GEPIA | <http://gepia.cancer-pku.cn/> | The Gene Expression Profiling Interactive Analysis (GEPIA) database is an interactive web that includes 9,736 tumors and 8,587 normal samples from TCGA and the GTEx projects. GEPIA was used to generate survival curves, including overall survival (OS) and Disease-free survival (DFS), based on gene expression with the logrank test and the Mantel-Cox test in colorectal cancer. |
| c-BioPortal | [http://cbioportal.org](http://cbioportal.org/) | The cBio Cancer Genomics Portal has multidimensional cancer genomics data sets. Mutation, copy number variation (CNV), and gene cooccurrence of ITGB3 in COAD were analyzed using the c-BioPortal tool. The tab OncoPrint displays an overview of genetic alterations per sample in ITGB3. |
| Networkanalyst | <https://www.networkanalyst.ca/> | Network interpreting gene expression was used by NetworkAnalyst 3.0 tool, which integrates cell-type or tissuespecific protein-protein interaction (PPI) networks, gene regulatory networks, and gene co-expression networks. |
| TIMER | <https://cistrome.shinyapps.io/timer/> | TIMER is a comprehensive resource for systematic analysis of immune infiltrates across diverse cancer types from TCGA,which includes 10,897 samples across 32 cancer types. TIMER applies a deconvolution method to infer the abundance of tumor-infiltrating immune cells (TIICs) from gene expression profiles. We analyzed ITGB3 expression in COAD and the correlation of ITGB3 expression with the abundance of immune infiltrates, including B cells, CD4+ T cells, CD8+ T cells, neutrophils, macrophages, and dendritic cells, as well as the tumor purity. |
| Immport | <https://www.immport.org/home> | ImmPort is an open access platform for sharing research data, expanding the value of scientific data in all fields of immunology, advancing hypothesis-driven and hypothesis-generating research, and providing analytical tools for advancing basic and clinical immunology research. |
| String | <https://string-db.org/> | The STRING database is an online search for known protein interaction relationships. It has been updated to version 11.0. It stores 2031 species, 9,643,763 proteins, and 1,380,838,440 interaction information. |

Supplementary Table 2. Functional roles of three seed genes and ten hub genes.

| Class | No. | Gene symbol | Full name | Function |
| --- | --- | --- | --- | --- |
| Seed genes | 1 | TPM2 | Tropomyosin 2 | Pathways: Dilated cardiomyopathy (DCM) and Cardiac muscle contraction; GO: Actin binding and structural constituent of muscle.Studies confirm that the TPM2 mRNA expression in CRC tissues is significantly lower than that in paired paracancerous tissues.TPM2 is methylated in colon cancer and its expression is lost, while TPM2 can be negatively regulated by the RhoA signaling pathway. |
|  | 2 | GNAO1 | G Protein Subunit Alpha O1 | Pathways: Neuroscience and Translation Translation regulation by Alpha-1 adrenergic receptors; GO: GTP binding and obsolete signal transducer activity. GNAO1 is overexpressed in gastric cancer and that its overexpression correlates with poor prognosis, as it promotes gastric cancer cell viability. |
|  | 3 | CHRDL1 | Chordin Like 1 | Pathways: [Signaling by GPCR](http://pathcards.genecards.org/card/signaling_by_gpcr) and [Signaling by BMP](http://pathcards.genecards.org/card/signaling_by_bmp).  CHRDL1 is a secreted protein and an antagonist of bone morphogenetic protein (BMP). As the activator of BMP receptor II (BMPRII), BMP mediates the signal transmission from outside the cell to the cell, and participates in the occurrence and metastasis of tumors. |
| Hub genes | 1 | ACTA2 | Actin Alpha 2, Smooth Muscle | Pathways: Development Slit-Robo signaling and Actin Nucleation by ARP-WASP Complex; GO: Protein kinase binding.ACTA2 is one of six different actin subtypes and is involved in smooth muscle contraction. ACTA2 is encoded by the ACTA2 gene located at 10q22-q24. Mutations in this gene cause a variety of vascular diseases, such as thoracic aortic disease, coronary artery disease, stroke, moyamoya disease, and multiple system smooth muscle dysfunction syndrome. ACTA2 (α-SMA) is currently considered a marker of myofibroblasts. |
|  | 2 | MYH11 | Myosin Heavy Chain 11 | Pathways: Signaling by GPCR and Pathogenic Escherichia coli infection; GO: Calmodulin binding and motor activity. Myosin heavy chain 11 (MYH11), encoded by the MYH11 gene, is a protein that participates in muscle contraction through the hydrolysis of adenosine triphosphate. Although previous studies have demonstrated that MYH11 gene expression levels are downregulated in several types of cancer |
|  | 3 | MYLK | Myosin Light Chain Kinase | Pathways: Association Between Physico-Chemical Features and Toxicity Associated Pathways and Signaling by GPCR; GO: transferase activity, transferring phosphorus-containing groups and protein tyrosine kinase activity. MYLK is mainly composed of smooth muscle myosin light chain kinase smMYLK and non-muscle myosin light chain kinase nmMYLK, both of which are indispensable participants in biological processes.It has been reported that the expression changes of MYLK is closely related to the occurrence and development of gastric cancer and colon cancer. |
|  | 4 | MYL9 | Myosin Light Chain 9 | Pathways: Guidance Cues and Growth Cone Motility and Signaling by GPCR; GO: Calcium ion binding and structural constituent of muscle.It has been reported that the expression changes of MYL9 is closely related to the occurrence and development of gastric cancer and colon cancer. |
|  | 5 | ACTG2 | Actin Gamma 2 | Pathways: Development Slit-Robo signaling and Actin Nucleation by ARP-WASP Complex. ACTG2 is up-regulated in aggressive tumors such as prostate cancer, colorectal cancer, breast cancer, pancreatic cancer, testicular cancer, and oral squamous cell carcinoma.Actins are highly conserved proteins that are involved in various types of cell motility and in the maintenance of the cytoskeleton.This gene encodes actin gamma 2; a smooth muscle actin found in enteric tissues. |
|  | 6 | CALD1 | Caldesmon 1 | Pathways: Myometrial Relaxation and Contraction Pathways and Cardiac conduction; GO:actin binding and myosin binding. This gene encodes a calmodulin- and actin-binding protein that plays an essential role in the regulation of smooth muscle and nonmuscle contraction. The conserved domain of this protein possesses the binding activities to Ca(2+)-calmodulin, actin, tropomyosin, myosin, and phospholipids. This protein is a potent inhibitor of the actin-tropomyosin activated myosin MgATPase, and serves as a mediating factor for Ca(2+)-dependent inhibition of smooth muscle contraction |
|  | 7 | LMOD1 | Leiomodin 1 | Pathways: Cardiac conduction and Smooth Muscle Contraction;GO:Actin binding and tropomyosin binding.The leiomodin 1 protein has a putative membrane-spanning region and 2 types of tandemly repeated blocks. The transcript is expressed in all tissues tested, with the highest levels in thyroid, eye muscle, skeletal muscle, and ovary. Increased expression of leiomodin 1 may be linked to Graves' disease and thyroid-associated ophthalmopathy |
|  | 8 | TAGLN | Transgelin | GO:Actin binding and actin filament binding. Interaction between TAGLN and HMGA2 was involved in TGF-β-induced cell migration and promotion of colon cancer cells. The expressions of TAGLN were inhibited in colorectal carcinoma tissues and colorectal carcinoma LoVo cells. The study also demonstrated that TAGLN could attenuate the proliferation and invasive ability of LoVo cells and enhance LoVo cell apoptosis. Furthermore, the expression of MMP9 was also inhibited by TAGLN. |
|  | 9 | CNN1 | Calponin 1 | Pathways:Mesenchymal Stem Cells and Lineage-specific Markers and Myometrial Relaxation and Contraction Pathways; GO:Actin binding and calmodulin binding.TAGLN is highly expressed in CRC tissues and cells. Silencing TAGLN can inhibit the proliferation, invasion and migration of CRC cells, suggesting that TAGLN plays an important role in the occurrence and development of CRC. |
|  | 10 | TPM2 | Tropomyosin 2 | Pathways: Dilated cardiomyopathy (DCM) and Cardiac muscle contraction; GO:Actin binding and structural constituent of muscle. TPM2 mRNA expression in CRC tissues is significantly lower than that in paired paracancerous tissues. Immunohistochemical analysis confirms that TPM2 is almost undetectable at the protein level in CRC tissues. Basic research shows that TPM2 is methylated in colon cancer and its expression is lost, while TPM2 can be negatively regulated by the RhoA signaling pathway. Therefore, loss of TPM2 expression can stimulate tumor proliferation by activating the RhoA pathway. |

Supplementary Table 3. The top 8 DEGs co-expressed miRNAs of ITGβ3 of in COAD samples( Cor>0.5)

| Term | Cor | P | Description |
| --- | --- | --- | --- |
| mir-143 | 0.60 | 4.49E-45 | The expression level of miR-143 in the cancer group was lower than that in the colonic mucosa group. Mir-143 can be control Smad pathway to affect the migration and invasion ability of CRC cells. |
| mir-133a | 0.58 | 7.21E-42 | MiR-133a is significantly downregulated in malignant cancers and often accompanied by poor prognosis. It also participates in various biological processes, including proliferation, apoptosis, autophagy, migration, invasion and drug resistance, which rely on the regulation of downstream target genes and signaling pathways, with the EGFR/ c-Myc/P53 axis as the commonly seen one. |
| mir-132 | 0.56 | 2.89E-39 | miR-132 is the target of cAMP response element-binding protein (CREB). It has been shown to regulate the growth of blood vessels, regulate neurodegeneration and differentiation of dopamine neurons, and play a role in controlling inflammation.miR-132 may inhibit tumor migration by down-regulating the expression of G3BP1/G3BP2. |
| mir-140 | 0.55 | 9.12E-37 | miR-140 is located on human chromosome 16q22.1, which is a fragile part of the chromosome . The study found that miR-140 is located in the intron of E3 ubiquitin ligase WWP2. Mature miR-140 is co-expressed with the spliceosome gene of WWP2-C and is regulated by its promoter. Overexpression of miR-140-5p can significantly inhibit the growth of CSC and the formation of stem cell spheres, while in vivo studies have found that overexpression of miR-140-5p can inhibit the tumorigenesis and metastasis of colon cancer CSC. |
| mir-139 | 0.54 | 1.49E-35 | miR-139 regulates diverse biological processes,such as proliferative, proliferation, invasion, metastasis, and progesterone receptor signaling pathways, promotes cell death by tumor suppressors and participates miRNA-regulated PINs, |
| mir-125a | 0.54 | 2.30E-35 | miR-490 is located on chromosome 7 and is abnormally expressed in many tumors. The targets of miR-490 involve a range of cancer-related genes involved in the regulation of various cancer hallmarks like cell proliferation, migration, and invasion, apoptotic cell death, angiogenesis. Recent studies have found that 90% of colorectal cancer patients have significantly down-regulated miR-490-5p levels in tumor tissues. |
| let-7c | 0.52 | 1.31E-32 | In addition to inhibiting tumor metastasis, the Let-7 family has been shown to affect tumor chemotherapy and radiotherapy sensitivity. More generally, the Let-7 family plays a role in the development of poorly differentiated malignant tumors. c-Myc is one of the target genes of let-7c. Let-7c works by binding to the complementary binding site of c-Myc MRNA 3 UTR, directly regulating the expression of c-Myc, thereby regulating the proliferation and apoptosis of tumor cells. |
| let-7e | 0.50 | 5.16E-30 | MicroRNA let-7 regulates stem cell differentiation, regulates development, and inhibits tumorigenesis and development. These functions are closely related to the expression level of let-7. Let-7e can play an extremely important biological role in embryonic stem cell differentiation, tumor development, nervous system function, and inflammatory response by regulating the expression of its mRNA target genes. |

Supplementary Table 4. Correlation analysis between ITGβ3 and Immune-Related Genes.

| Category | Gene Symbol | Gene Name | None | | Purity | |
| --- | --- | --- | --- | --- | --- | --- |
|  |  |  | Cor | P | Cor | P |
| Antimicrobials | AHNAK | AHNAK nucleoprotein | 0.4540 | 1.13E-24 | 0.4207 | 7.58E-19 |
|  | AQP9 | Aquaporin 9 | 0.5851 | 2.06E-43 | 0.5489 | 2.54E-33 |
|  | CCL21 | C-C motif chemokine ligand 21 | 0.6572 | 5.78E-58 | 0.5892 | 2.66E-39 |
|  | CCR1 | C-C motif chemokine receptor 1 | 0.2613 | 1.38E-08 | 0.6364 | 1.76E-47 |
|  | CSRP1 | Cysteine and glycine rich protein 1 | 0.6826 | 4.27E-64 | 0.5288 | 1.27E-30 |
|  | CXCL12 | C-X-C motif chemokine ligand 12 | 0.6380 | 1.03E-53 | 0.6429 | 1.02E-48 |
|  | CYBB | Cytochrome b-245 beta chain | 0.5446 | 9.95E-37 | 0.6121 | 4.15E-43 |
|  | DES | Desmin | 0.6080 | 1.24E-47 | 0.5298 | 9.27E-31 |
|  | LTBP1 | Latent transforming growth factor beta binding protein 1 | 0.5580 | 7.59E-39 | 0.5290 | 1.17E-30 |
|  | MARCO | Macrophage receptor with collagenous structure | 0.4985 | 3.85E-30 | 0.4448 | 4.04E-21 |
|  | MASP1 | Mannan binding lectin serine peptidase 1 | 0.6760 | 1.95E-62 | 0.6595 | 5.02E-52 |
|  | MMP9 | Matrix metallopeptidase 9 | 0.6174 | 1.78E-49 | 0.5707 | 1.82E-36 |
|  | OLR1 | Oxidized low density lipoprotein receptor 1 | 0.5332 | 5.30E-35 | 0.4772 | 1.78E-24 |
|  | PTGS2 | Prostaglandin-endoperoxide synthase 2 | 0.4574 | 4.58E-25 | 0.4143 | 2.85E-18 |
| BCRSignalingPathway | AKT3 | AKT serine/threonine kinase 3 | 0.7744 | 1.10E-92 | 0.7517 | 5.01E-75 |
|  | NFAT5 | Nuclear factor of activated T cells 5 | 0.2182 | 2.43E-06 | 0.2338 | 1.91E-06 |
| Chemokine_Receptors | CMKLR1 | Chemerin chemokine-like receptor 1 | 0.7208 | 1.32E-74 | 0.6451 | 3.71E-49 |
|  | EDNRA | Endothelin receptor type A | 0.5632 | 1.09E-39 | 0.5671 | 6.27E-36 |
|  | PLXNC1 | Plexin C1 | 0.6747 | 3.94E-62 | 0.6502 | 3.73E-50 |
| Chemokines | SEMA3G | Semaphorin 3G | 0.5621 | 1.63E-39 | 0.5220 | 9.30E-30 |
|  | SEMA4G | Semaphorin 4G | 0.0826 | 7.72E-02 | 0.0811 | 1.03E-01 |
| Cytokine_Receptors | ANGPTL2 | Angiopoietin like 2 | 0.7561 | 5.52E-86 | 0.7299 | 9.85E-69 |
|  | CALCRL | Calcitonin receptor like receptor | 0.7061 | 2.30E-70 | 0.6798 | 2.34E-56 |
|  | CSF1R | Colony stimulating factor 1 receptor | 0.6958 | 1.55E-67 | 0.6977 | 1.67E-60 |
|  | CSF2RB | Colony stimulating factor 2 receptor subunit beta | 0.5956 | 2.61E-45 | 0.6805 | 1.59E-56 |
|  | CSF3R | Colony stimulating factor 3 receptor | 0.5592 | 4.84E-39 | 0.5751 | 4.07E-37 |
|  | EDNRB | endothelin receptor type B | 0.6562 | 9.90E-58 | 0.5548 | 3.74E-34 |
|  | FGFR1 | fibroblast growth factor receptor 1 | 0.1769 | 1.42E-04 | 0.7319 | 2.74E-69 |
|  | GCG | glucagon receptor | 0.6662 | 4.61E-60 | 0.1417 | 4.22E-03 |
|  | IL10RA | interleukin 10 receptor subunit alpha | 0.6331 | 1.14E-52 | 0.6010 | 3.16E-41 |
|  | IL1R1 | interleukin 1 receptor type 1 | 0.7477 | 4.20E-83 | 0.7308 | 5.58E-69 |
|  | IL6R | interleukin 6 receptor | 0.5367 | 1.60E-35 | 0.4993 | 5.49E-27 |
|  | KDR | kinase insert domain receptor | 0.7279 | 9.57E-77 | 0.7024 | 1.27E-61 |
|  | LIFR | LIF receptor subunit alpha | 0.5983 | 8.10E-46 | 0.5624 | 3.05E-35 |
|  | NR3C1 | nuclear receptor subfamily 3 group C member 1 | 0.6922 | 1.37E-66 | 0.6588 | 6.97E-52 |
|  | NR4A3 | nuclear receptor subfamily 4 group A member 3 | 0.5222 | 2.11E-33 | 0.4766 | 2.04E-24 |
|  | NRP1 | neuropilin 1 | 0.7522 | 1.18E-84 | 0.7207 | 2.94E-66 |
|  | NRP2 | neuropilin 2 | 0.7258 | 4.31E-76 | 0.6891 | 1.80E-58 |
|  | OSMR | oncostatin M receptor | 0.8231 | 4.04E-114 | 0.8072 | 1.61E-94 |
|  | RORA | RAR related orphan receptor A | 0.6024 | 1.40E-46 | 0.5734 | 7.40E-37 |
|  | S1PR1 | sphingosine-1-phosphate receptor 1 | 0.7970 | 6.69E-102 | 0.7750 | 1.54E-82 |
| Cytokines | BMP3 | bone morphogenetic protein 3 | 0.4667 | 3.81E-26 | 0.4302 | 1.01E-19 |
|  | C3 | complement C3 | 0.5658 | 4.13E-40 | 0.5183 | 2.69E-29 |
|  | CHGA | chromogranin A | 0.0926 | 4.75E-02 | 0.2221 | 6.25E-06 |
|  | FGF7 | fibroblast growth factor 7 | 0.7594 | 3.78E-87 | 0.6158 | 9.42E-44 |
|  | GREM1 | gremlin 1, DAN family BMP antagonist | 0.4846 | 2.41E-28 | 0.6359 | 2.14E-47 |
|  | GREM2 | gremlin 2, DAN family BMP antagonist | 0.1445 | 1.93E-03 | 0.4490 | 1.56E-21 |
|  | GUCA2A | guanylate cyclase　activator 2A | 0.1445 | 1.93E-03 | 0.1066 | 3.18E-02 |
|  | LTBP2 | latent transforming growth factor beta binding protein 2 | 0.7073 | 1.08E-70 | 0.6693 | 4.44E-54 |
|  | OGN | osteoglycin | 0.4919 | 2.81E-29 | 0.4778 | 1.53E-24 |
|  | PDGFC | platelet derived growth factor C | 0.5990 | 6.15E-46 | 0.5558 | 2.76E-34 |
|  | PDGFRA | platelet derived growth factor receptor alpha | 0.6936 | 5.90E-67 | 0.6723 | 9.94E-55 |
|  | PDGFRB | platelet derived growth factor receptor beta | 0.7370 | 1.33E-79 | 0.7005 | 3.70E-61 |
|  | PYY | peptide YY | 0.1297 | 5.45E-03 | 0.0627 | 2.08E-01 |
|  | RETNLB | resistin like beta | -0.0826 | 7.75E-02 | -0.0753 | 1.30E-01 |
| Interleukins | IL6ST | interleukin 6 signal transducer | 0.7521 | 1.33E-84 | 0.7426 | 2.55E-72 |
| Interleukins_Receptor | IL7R | interleukin 7 receptor | 0.6259 | 3.53E-51 | 0.6006 | 3.68E-41 |
| NaturalKiller_Cell_Cytotoxicity | LCP2 | lymphocyte cytosolic protein 2 | 0.6252 | 4.81E-51 | 0.6013 | 2.87E-41 |
|  | PIK3R5 | phosphoinositide-3-kinase regulatory subunit 5 | 0.6622 | 4.03E-59 | 0.6343 | 4.32E-47 |
| TCRsignalingPathway | CHP2 | calcineurin like EF-hand protein 2 | 0.6770 | 1.06E-62 | 0.0946 | 5.69E-02 |
| TGFb_Family_Member | INHBA | inhibin subunit beta A | 0.7267 | 2.30E-76 | 0.6900 | 1.14E-58 |
|  | LEFTY1 | left-right determination factor 1 | -0.0646 | 1.68E-01 | -0.0655 | 1.88E-01 |


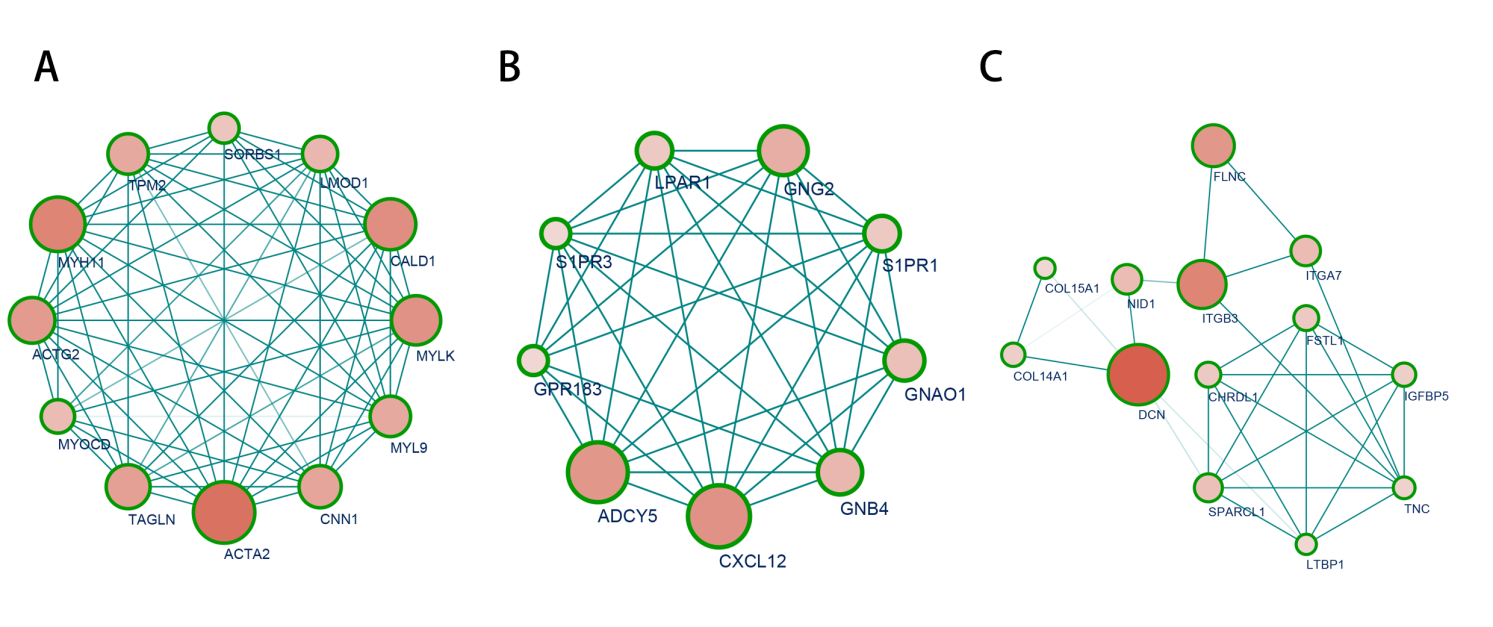


**Supplementary Figure 1.** The PPI network of three most significant modules.


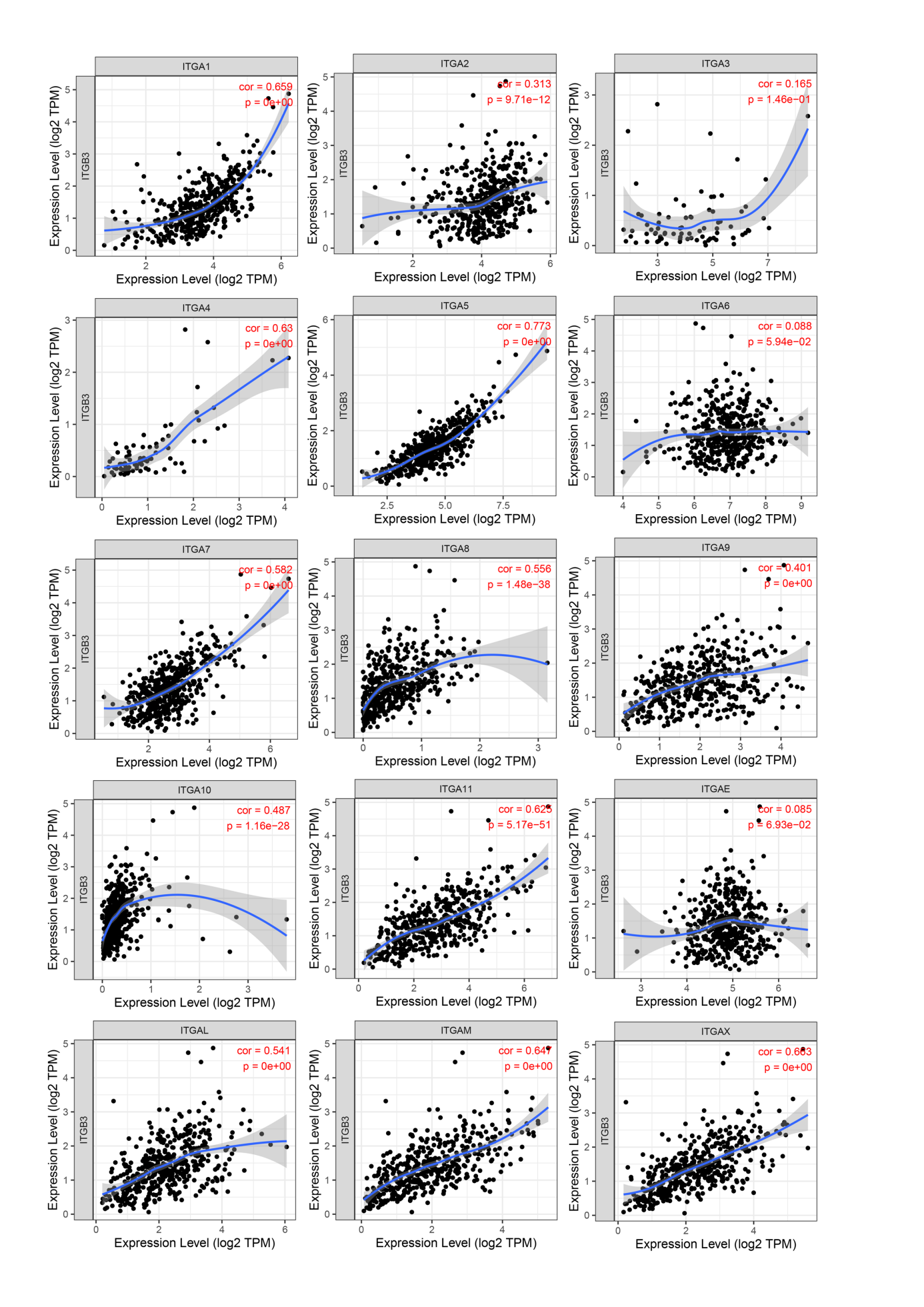


**Supplementary Figure 2.** Correlation analysis between ITGβ3 and ITGα genes.
